# Outcome of Orchidopexy in Spigelian Hernia-Undescended Testis Syndrome

**DOI:** 10.7759/cureus.13714

**Published:** 2021-03-05

**Authors:** Abdulrahman Taha, Nada E Algethami, Raghad AlQurashi, Amal K Alnemari

**Affiliations:** 1 Pediatric Surgery, Raparin Teaching Hospital for Children, Erbil, IRQ; 2 Medicine, Taif University, Taif, SAU; 3 Medicine, Al-Hada Governmental Hospitals, Taif, SAU

**Keywords:** spiegel hernia, undescended testis, congenital, abdominal wall defect, children

## Abstract

Spigelian hernia-undescended testes (SH-UDT) syndrome is a rare disorder, with only 57 cases reported in the literature. The presentation can be asymptomatic or symptomatic in the form of pain, tenderness, or a lump. We present a case of a 50-day-old boy with SH-UDT syndrome. The patient presented with signs and symptoms of acute intestinal obstruction. Exploration confirmed a Spigelian hernia containing small bowel loops and right undescended testis. Orchidopexy was done after ligation of the hernial sac. A follow-up visit after two years revealed right testicular atrophy.

## Introduction

Spigelian hernia (SH) is a rare condition with just 57 cases reported in the literature among those younger than 15 years old [1]. SH is a type of ventral hernia, described as a protrusion of any intraabdominal contents through the abdominal wall along the semilunar line, particularly at the semilunar junction and the arcuate line. SHs account for approximately 2% of all ventral hernias and less than 1% of all abdominal hernias [2]. The most common symptom is pain, but the clinical presentation differs depending on the content of the hernial sac, the level, and the degree of herniation [3]. SH is associated with ipsilateral cryptorchidism in about 28%-75% of male pediatric patients. There are few resources in the literature discussing the outcome of orchidopexy in Spigelian hernia-undescended testes (SH-UDT) syndrome.

## Case presentation

A 50-day-old boy, a member of a triplet pregnancy, presented with abdominal distension, bile-stained vomiting, and constipation. His condition was preceded by a few days of frequent bowel motion.

**Figure 1:**
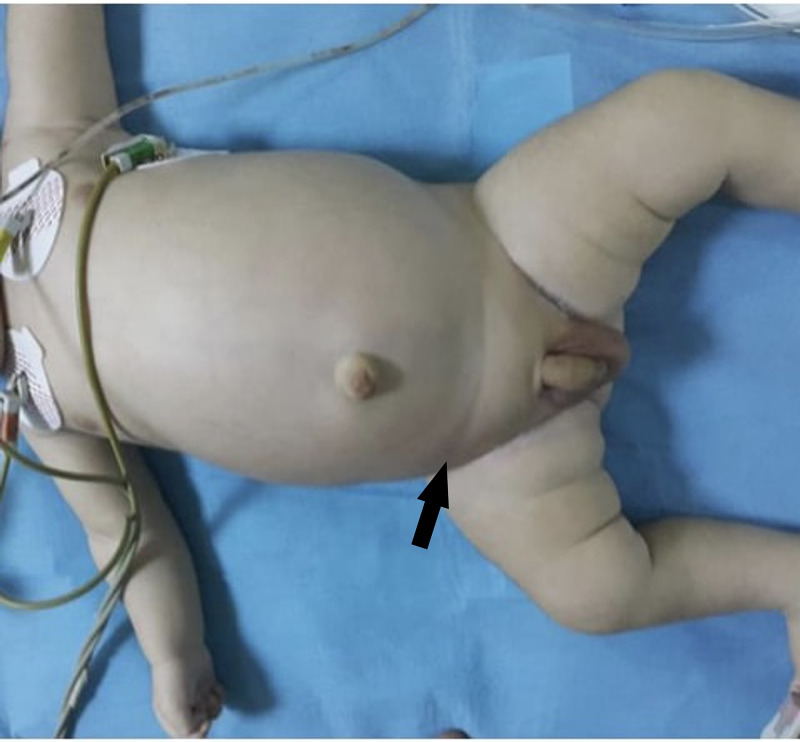
Preoperative picture showing abdominal distention, empty right hemiscrotum and right lower quadrant lump seen by inspection.

**Figure 2:**
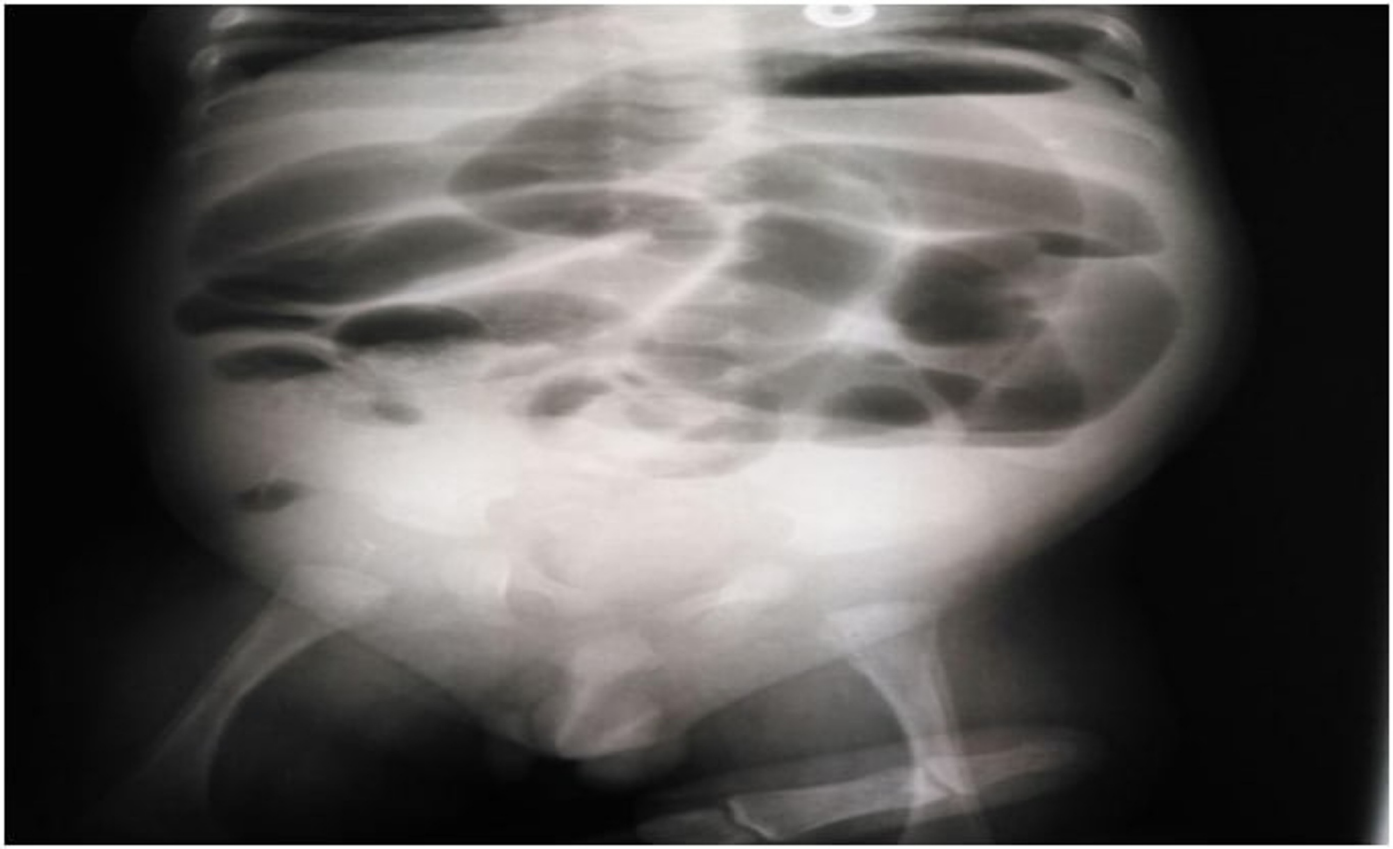
AP supine abdominal radiograph.

On examination, the baby was irritable, mildly dehydrated, with moderate abdominal distension, on palpation, the abdomen was tense with a mass and tenderness in the right lower quadrant region. The right testis was not palpable, neither in the scrotum nor in the inguinal region (Figure 1). After fluid resuscitation, the patient was sent for basic investigations. A plain supine abdominal radiograph showed distended bowel loops, gasless lower abdomen, and right lower quadrant lucency (Figure 2).

The abdominal US showed a small amount of intraperitoneal free fluid and a loop of bowel (25 × 16 mm) herniated through the abdominal wall, defect; the defect was (10 mm) in diameter.

**Figure 3:**
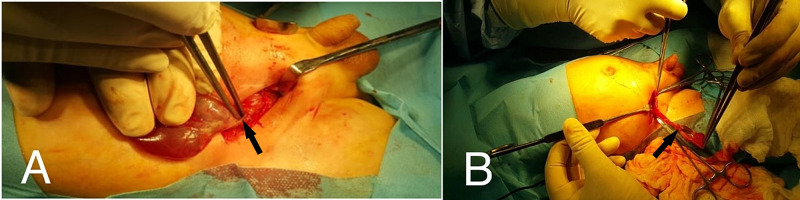
Intraoperative picture. A: Herniated sac through the lateral wall defect. B: Separation of cord structures from the sac and ligate the sac, right testis looks bluish in color.

The decision was made to perform open surgery. An incision was made over a small lump in the right lower quadrant region, cystic fluid containing a sac was found herniated lateral to the rectus abdominis muscle communicating with a peritoneal cavity by a small defect between muscle layers. When the sac was opened, it contained fluid, loops of the small bowel and the right testis. The hernia defect was widened, the healthy bowel reduced, the testis and cord structures separated from the posterior wall of the sac, the sac ligated, after confirmation of adequate length of the testis to scrotum extraabdominaly, a tract created between the right scrotal incision and abdominal incision extraperitoneal, medial to the lower epigastric vessels, and the testis delivered to the right hemiscrotum and subdartos orchidopexy (Figure 3).

**Figure 4:**
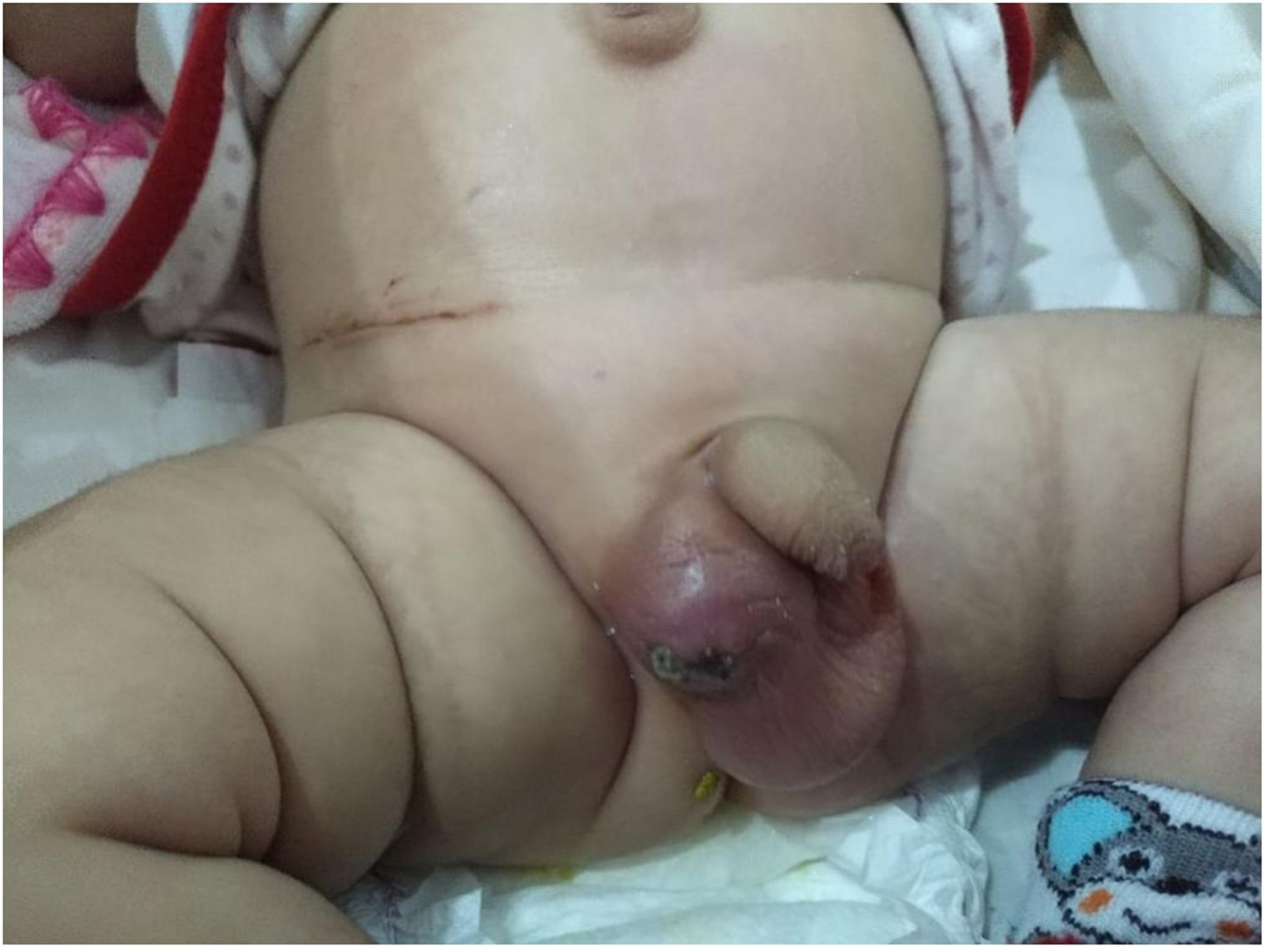
One-week post-op picture showing scar in the right lower quadrant, right testes in the scrotum with signs of infection.

Postoperative recovery was smooth. The patient was discharged after 72 hours; a follow-up visit after one week revealed a stable patient, no abdominal distension, and with the right testis was in the right hemiscrotal position, but the scrotal skin showed signs of infection, tenderness, and erythematous changes, the patient was on oral and local antibiotics postoperatively (Figure 4). The patient missed follow-up for more than two years, and on the last visit, we found that no right testis was felt in the scrotum, and it was atrophied.

## Discussion

In 1895, the first case of SH with ipsilateral UDT was reported by Schoofs [4]. This type is uncommon, with a frequency of about 1%-2% [5]. The cause can be acquired or congenital, and as in the pediatric group, the causes remain unclear, and many hypotheses have been put forward. For example, alterations in the structure of the transverses abdominis and internal oblique muscles with the developmental aberration of abdominal muscle and muscular paralysis [6,7].

There is debate among researchers about whether UDT is a primary defect and SH occurs secondary, or vice versa or the primary cause is the failure to develop gubernaculum [8]. Raveenthiran and colleagues proposed that the ectopic position of the testis is the primary cause, leading to SH [9,10]. Rushfeldt et al. attributed the failure to develop a gubernaculum to arrested (intra-abdominal) testis development, failing to descend to the normal position in the scrotum [11]. The average age for congenital SH presentation is 4.52 years but ranges from newborns to 17-year-olds [7]. A baby with reducible anterior abdominal wall swelling and UDT should increase the clinician’s suspicion [5]. Presentation varies from asymptotic to symptomatic (localized abdominal pain and bulge along the lateral border of rectus abdominis, intermittently) [12]. The rarity of SH may delay its diagnosis. Only 50% of cases are diagnosed preoperatively [7,12]. The use of ultrasonography as the first imaging modality preoperatively can help establish the correct diagnosis [8,12].

Management of SH is surgical through open technique or laparoscopic modalities [7,13-15]. The laparoscopy is considered a new approach according to three cases reported by Kumar et al., Desmukh et al., and Khan et al. [12,16,17]. As our case presented with signs of acute intestinal obstruction, we preferred to do an open surgery to manage the case; after opening the sac, which contained fluid, small bowel loops, and the testis, the bowel was healthy, but the testis looked ischaemic. The abdominal wall defect was widened, the bowel reduced, the cord structures separated from the sac and the sac ligated, a tract was made between the abdomen and right hemiscrotum medial to epigastric vessels, and after making the scrotal incision, the testis was delivered to the right scrotum, and subdartos orchidopexy was done with moderate tension. There is little literature discussing the outcome of orchidopexy in SH-UDT syndrome.

Dr. Inan reported that it is possible to avoid damage of testes by performed surgical repair of congenital SH in the early period, and orchidopexy could be postponed to one year of age, when the risk of incarceration is decreased. The testis and spermatic cord are enlarged, and the infant’s immune response is getting stronger. Possible factors of testicular atrophy in our case: the testis was ischemic at the time of presentation, orchidopexy was done early in life, and orchidopexy was done under moderate tension [18].

We suspect the infection was the result of dead testicular tissue rather than the cause of atrophy. Also, we believe that the new canal was not the cause of atrophy, as this is routinely done for intraabdominal testis without a high rate of atrophy. We support the idea of repairing the hernia and delaying orchidopexy in SH-UDT syndrome after one year of age to decrease the risk of testicular atrophy.

## Conclusions

The association between SH and UDT is well documented; when present during infancy; the hernia should be repaired after reduction of the testis into the abdominal cavity and orchidopexy should be postponed to near one year of age to decrease damage to fragile testicular tissue especially in case of emergency situations.
